# Kinetic Analysis of the Hydrolysis of Pentose‐1‐phosphates through Apparent Nucleoside Phosphorolysis Equilibrium Shifts**

**DOI:** 10.1002/cphc.202000901

**Published:** 2020-12-04

**Authors:** Felix Kaspar, Peter Neubauer, Anke Kurreck

**Affiliations:** ^1^ Chair of Bioprocess Engineering Technische Universität Berlin Straße des 17. Juni 135 10632 Berlin Germany; ^2^ BioNukleo GmbH Ackerstraße 76 13355 Berlin Germany

**Keywords:** decay, phosphate, ribose, sugar phosphate, UV spectroscopy

## Abstract

Herein, we report an addition to the toolbox for the monitoring and quantification of the hydrolytic decay of pentose‐1‐phosphates, which are known to be elusive and difficult to quantify. This communication describes how apparent equilibrium shifts of a nucleoside phosphorolysis reaction can be employed to calculate hydrolytic loss of pentose‐1‐phosphates based on the measurement of post‐hydrolysis equilibrium concentrations of a nucleoside and a nucleobase. To demonstrate this approach, we assessed the stability of the relatively stable ribose‐1‐phosphate at 98 °C and found half‐lives of 1.8–11.7 h depending on the medium pH. This approach can be extended to other sugar phosphates and related reaction systems to quantify the stability of UV‐inactive and hard‐to‐detect reaction products and intermediates.

## Graphical abstract

**Ask what an equilibrium can do for you**: Hydrolysis of pentose‐1‐phosphates leads to an apparent shift of the equilibrium conversion in nucleoside phosphorolysis reactions. This information can be leveraged via equilibrium thermodynamics to determine the hydrolysis kinetics of *in situ* generated sugar phosphates.

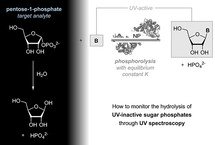

Many biomolecules of the central metabolism are rather elusive, owed to their nature as highly polar UV‐inactive small molecules.[^1^, ^2^, ^3^] Prominent examples include non‐aromatic amino acids (building blocks of proteins), sugar phosphates (intermediates of glycolysis and nucleoside catabolism) and small carboxylic acids (intermediates of amino acid metabolism and Krebs cycle). Nonetheless, research of cellular processes as well as enzymatic reactions, both *in vivo* and *in vitro*, requires a fundamental quantitative understanding of the turnover and the stability of these molecules. Where common high‐throughput HPLC‐based detection of analytes fails, sensitive GC‐based methods, mass spectrometry, derivatization of target molecules (or a combination of the above) or detection via coupled fluoro‐ or chromogenic reactions can serve as alternatives. Detection and quantification via NMR, TLC, polarimetry, refractometry or specific enzymatic assays might also be employed, depending on the target analyte. However, many of these methods typically suffer from limited throughput and, often, a narrow working space. Furthermore, detection from complex mixtures, such as enzymatic reactions or buffered aqueous solutions, can prove difficult or impossible with some of the above methods.

Herein, we report an addition to the toolbox for the monitoring and quantification of the hydrolytic decay of pentose‐1‐phosphates. These compounds are central to nucleoside catabolism, nucleoside/nucleotide salvage pathways as well as the pentose phosphate pathway.^[4]^ Further, they serve as synthetic intermediates and sugar donors in nucleoside phosphorylase‐catalyzed transglycosylations.[^5^, ^6^, ^7^] Their synthesis typically occurs via phosphorolytic cleavage of a nucleoside, liberating the corresponding nucleobase and pentose‐1‐phosphate (Figure 1A). Once generated, pentose‐1‐phosphates are valuable intermediates as they allow direct glycosylation of (nearly) any other nucleobase. Thus, irreversible hydrolysis of these sugar synthons represents a costly loss – both *in vivo* and in synthesis.[^8^, ^9^, ^10^, ^11^] Previous work from Bunton and Humeres^[12]^ had employed a colorimetric phosphate assay and work from our lab^[13]^ had used TLC for a preliminary screening of pentose‐1‐phosphate stability. Both studies revealed that acidic conditions as well as high temperatures promote hydrolysis. However, painting of a clearer picture requires more sensitivity and throughput than TLC or colorimetric phosphate detection from diluted mixtures offer. Furthermore, direct analysis of pure pentose‐1‐phosphates is discouraged by their limited commercial availability and deterring price point (ca. 50 € mg^−1^ for ribose‐ and 2‐deoxyribose‐1‐phosphate as of September 2020).

**Figure 1:**
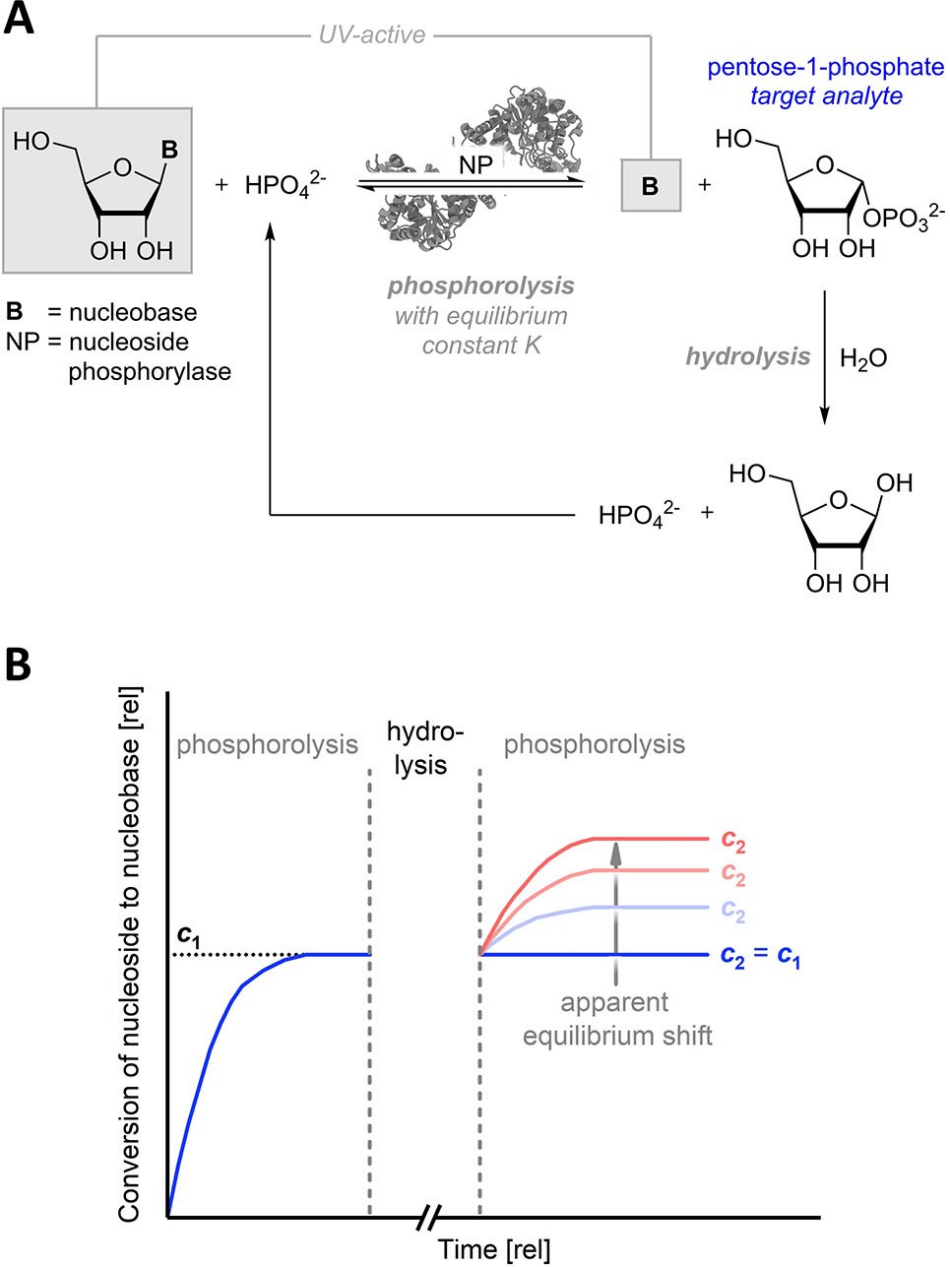
Enzymatic reversible phosphorolysis of a nucleoside with subsequent irreversible hydrolysis of the product pentose‐1‐phosphate (**A**) and illustrative reaction time course of a discontinuous performance of phosphorolysis and hydrolysis (**B**). $c_{1}$ and $c_{2}$ are the degrees of conversion from the nucleoside to the nucleobase in the pre‐ and post‐hydrolysis equilibria as defined below. Phosphate liberated by hydrolysis of the target analyte feeds back into the substrate reservoir *in situ*. Monitoring of the phosphorolysis reaction can be achieved via the two UV‐active components (nucleoside and nucleobase).[^15^, ^16^] The graphs shown in **B** only serve an illustrative purpose and do not represent real data.

This report details how apparent equilibrium shifts of nucleoside phosphorolysis can be used to assess the hydrolysis of *in situ* generated pentose‐1‐phosphates. For this approach we relied on nucleoside phosphorolysis as a tightly thermodynamically controlled reaction with equilibrium constants in the range of 0.01–0.8,^[14]^ which allows one to calculate all reagent concentrations of an enzymatic reaction in equilibrium solely by measuring two UV‐active components (Figure 1A). We anticipated that knowledge of the equilibrium constant of a given transformation would enable us to quantify loss of one reagent (pentose‐1‐phosphate) via the mass balances off all reagents and a shifted apparent equilibrium position caused by the reaction system compensating the loss of a product via additional substrate consumption following Le Chatelier's principle. Having previously employed spectral unmixing‐based reaction monitoring to detect slight temperature‐dependent changes in the chemical equilibrium of many nucleoside phosphorolysis reactions,[^14^, ^15^, ^16^] we again turned to this method to record small spectral differences to quantify minor equilibrium shifts. To effect these equilibrium shifts, we let nucleoside phosphorolysis reactions reach a stable equilibrium, then subjected these mixtures to hydrolyzing conditions and, upon addition of fresh enzyme and continuation of phosphorolysis, allowed the reaction to establish a new equilibrium (Figure 1B). This incubation time‐dependent deviation of the new equilibrium from the old equilibrium could then be used to calculate the amount of lost pentose‐1‐phosphate.

The mathematical basis for this approach is given by the law of mass action,^[17]^ which describes the concentrations of all reaction components in a chemical equilibrium. In the case of nucleoside phosphorolysis, the concentrations of the nucleoside and nucleobase are easily accessible and the unknown concentrations of UV‐inactive components (phosphate and the sugar phosphate) are generally available through the assumption of stoichiometry and the derived mass balances.^[14]^ The equilibrium constant K of nucleoside phosphorolysis is defined as

(1) $$K=\frac{\left[P1P\right]\left[B\right]}{\left[N\right]\left[P\right]}$$

where $\left[P1P\right]$ , $\left[B\right]$ , $\left[N\right]$ and $\left[P\right]$ are the equilibrium concentrations of the pentose‐1‐phosphate, nucleobase, nucleoside and phosphate, respectively. Before hydrolysis to any significant degree has taken place, the following mass balances hold true for this reaction:

(2) $$\left[P1P\right]=\left[B\right]={\left[P1P\right]}_{1}={\left[B\right]}_{1}$$

(3) $$\left[N\right]={\left[N\right]}_{0}-{\left[B\right]}_{1}$$

(4) $$\left[P\right]={\left[P\right]}_{0}-{\left[B\right]}_{1}$$

where ${\left[N\right]}_{0}$ and ${\left[P\right]}_{0}$ are the starting (initial) concentrations of the nucleoside and phosphate and ${\left[P1P\right]}_{1}$ and ${\left[B\right]}_{1}$ are the concentrations of the pentose‐1‐phosphate and the nucleobase and the first (pre‐hydrolysis) equilibrium, respectively (assuming that the initial concentrations of both products, ${\left[P1P\right]}_{0}$ and ${\left[B\right]}_{0}$ , are equal to zero). The degree of conversion of the nucleoside to the nucleobase in this first equilibrium $c_{1}$ is given as

(5) $$c_{1}=\frac{{\left[B\right]}_{1}}{{\left[N\right]}_{0}}$$

After the establishment of an initial equilibrium, termination of the enzymatic reaction and subsequent hydrolysis of the pentose‐1‐phosphate the mass balances of the nucleoside and the nucleobase remain identical. However, the hydrolysis of some fraction of the sugar phosphate results in adjusted mass balances for this reagent and its hydrolysis product phosphate (and ribose, which is not part of this equilibrium and will therefore not be considered herein). At this point, addition of fresh nucleoside phosphorylase leads to further conversion of the nucleoside to equalize the loss of pentose‐1‐phosphate and reestablish reagent concentrations which fulfill the equilibrium constraint (1). The degree of conversion in this new equilibrium then reflects the amount of hydrolyzed pentose‐1‐phosphate (see the Supporting Information for more details and derivations). This results in the adjusted mass balances:

(6) $$\left[P1P\right]=c_{2}{\left[N\right]}_{0}-{\left[P1P\right]}_{h}$$

(7) $$\left[B\right]=c_{2}{\left[N\right]}_{0}$$

(8) $$\left[N\right]={\left[N\right]}_{0}-c_{2}{\left[N\right]}_{0}$$

(9) $$\left[P\right]={\left[P\right]}_{0}-c_{2}{\left[N\right]}_{0}+{\left[P1P\right]}_{h}$$

where ${\left[P1P\right]}_{h}$ is the amount of hydrolyzed pentose‐1‐phosphate and $c_{2}$ is the degree of conversion of the nucleoside to the nucleobase in the post‐hydrolysis equilibrium defined as

(10) $$c_{2}=\frac{{\left[B\right]}_{2}}{{\left[N\right]}_{0}}$$

where ${\left[B\right]}_{2}$ is the nucleobase concentration in the second (post‐hydrolysis) equilibrium. These mass balances then allow the calculation of the concentration of hydrolyzed sugar phosphate upon insertion into the law of mass action (1). Solving of the resulting expression for ${\left[P1P\right]}_{h}$ yields an equation which returns analytical solutions for known values of $c_{2}$ , ${\left[N\right]}_{0}$ , ${\left[P\right]}_{0}$ and K .

(11) $${\left[P1P\right]}_{h}=\frac{K\left({\left[P\right]}_{0}-c_{2}{\left[N\right]}_{0}-c_{2}{\left[P\right]}_{0}+c_{2}^{2}{\left[N\right]}_{0}\right)-c_{2}^{2}{\left[N\right]}_{0}}{Kc_{2}-c_{2}-K}$$

The expected maximum degree of conversion in the second equilibrium $c_{2,max}$ can be obtained through the assumption of full hydrolysis of the initially generated sugar phosphate ${(\left[P1P\right]}_{h}=c_{1}{\left[N\right]}_{0}$ ) and is given by the expression:

(12) $$c_{2,max}=\frac{-X}{2{\left[N\right]}_{0}\left(1-K\right)}+\frac{\sqrt{X^{2}+4\left({\left[N\right]}_{0}-K{\left[N\right]}_{0}\right)\left(c_{1}{\left[N\right]}_{0}K+K{\left[P\right]}_{0}\right)}}{2{\left[N\right]}_{0}\left(1-K\right)}$$

where

(13) $$X=\;c_{1}{\left[N\right]}_{0}K-c_{1}{\left[N\right]}_{0}+{\left[N\right]}_{0}K+K{\left[P\right]}_{0}$$

The incubation time‐dependent conversion $c_{2}$ is available directly through monitoring of the reaction at its second equilibrium, K can either be determined experimentally or obtained from the respective literature^[14]^ and the initial concentrations ${\left[N\right]}_{0}$ and ${\left[P\right]}_{0}$ are known from the experimental setup. Thus, equation (11) allows direct quantification of hydrolyzed pentose‐1‐phosphate solely by measuring post‐hydrolysis equilibrium positions of a nucleoside phosphorolysis reaction. Please note, however, that these equations assume that the equilibrium constant K can be determined with sufficient accuracy. Alternatively, replacement of K in (11) with the terms in equations (1) and (2) allows simultaneous determination of K and ${\left[P1P\right]}_{h}$ but demands monitoring of the first equilibrium via observation of the equilibrium conversion $c_{1}$ (also see the equations in^[14]^). Calculation of

(14) $${\Delta c}_{max}=c_{2,max}-c_{1}$$

then provides insight into the maximum apparent equilibrium shift ${\Delta c}_{max}$ as a function of ${\left[N\right]}_{0}$ , ${\left[P\right]}_{0}$ and K .

A theoretical examination of the solutions of this derived set of equations reveals a relatively broad operational window for equilibrium constants between 1 and 0.01 (Figure 2A). For the cheaply available sugar donor uridine (K =0.15 at 37 °C and pH 7.5),^[14]^ any phosphate to nucleoside ratio of 2.5–10 yields a potential equilibrium shift ${\Delta c}_{max}$ of >20 percentage points (pp; from the equilibrium position of no hydrolysis to the new equilibrium corresponding to full hydrolysis of the generated pentose‐1‐phosphate; Figure 2B), with the extreme ends of nucleoside conversion either generating almost no sugar phosphate ($c_{1}$ <10 %) or offering little space for equilibrium adjustment ($c_{1}$ >80 %).

**Figure 2:**
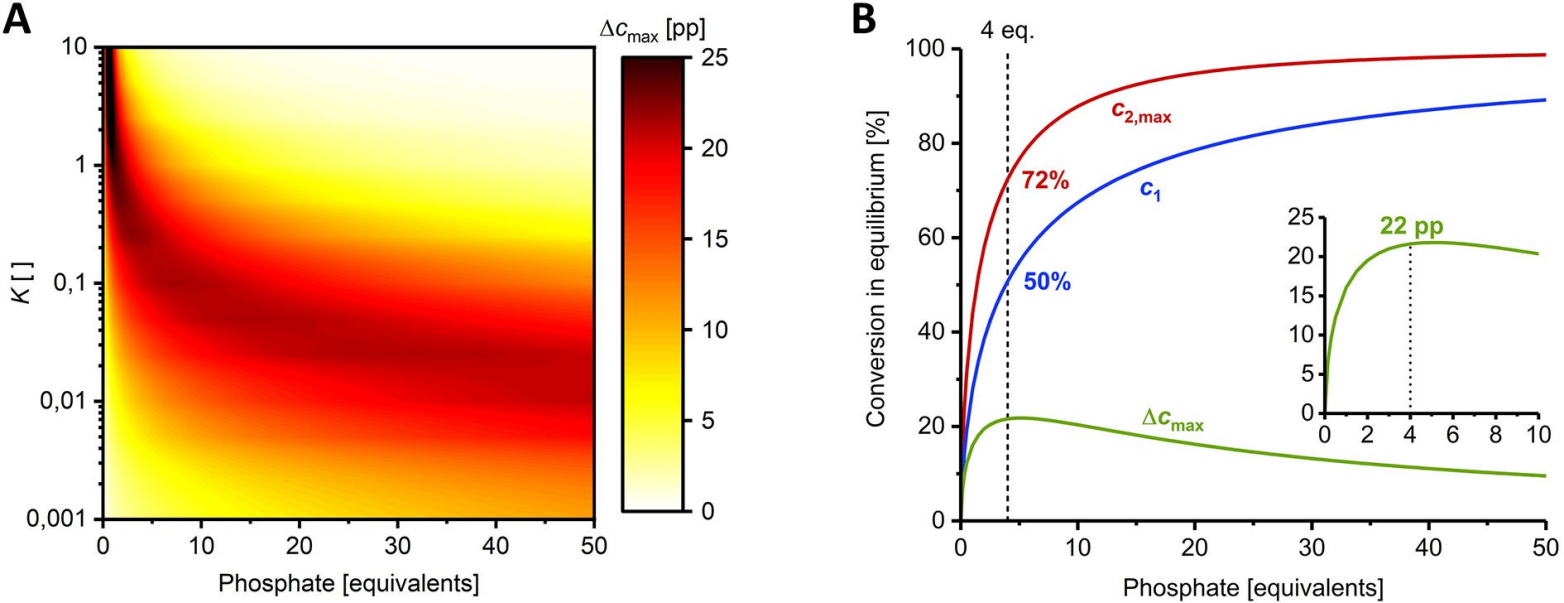
Working window for hydrolysis‐induced equilibrium shifts of nucleoside phosphorolysis. The working window should enable a maximum equilibrium shift ${\Delta c}_{max}$ as large as possible to permit accurate measurements of the effect. Suitable reaction conditions regarding phosphate excess depend on the equilibrium constant of the system (**A**). For uridine (K =0.15) the system is quite robust and offers a ${\Delta c}_{max}$ >20 pp over a broad phosphate excess range with an optimal zone of 4–6 equivalents (**B**). $c_{1}$ was calculated by solving equation (1) for the mass balances (2)–(4), as described previously,^[14]^ and $c_{2,max}$ and ${\Delta c}_{max}$ were calculated via equations (12) and (14) for different equilibrium constants. Please see the externally hosted Supplementary Information for the calculations.^[18]^

To demonstrate this method with a practical example, we assessed the hydrolysis kinetics of the relatively stable ribose‐1‐phosphate (**Rib1P**). While pentose‐1‐phosphates lacking the 2‐hydroxy group are prone to hydrolysis,^[13]^ ribosyl scaffolds offer much more stability.[^12^, ^13^] In fact, even high temperatures are known to effect only minimal decay of these molecules and previous work had largely limited their working space to acidic conditions – presumably to permit operation within a reasonable timeframe. To benchmark the stability of **Rib1P** at higher pH values, we generated this compound *in situ* and subjected it to pH values from 7–9 at a temperature of 98 °C. The application of 4 equivalents of phosphate (2 mM uridine, 8 mM phosphate), yielded a stable equilibrium of 50 % conversion to the corresponding nucleobase uracil and **Rib1P** (1 mM each at $c_{1}$ which agrees with previous reports).^[14]^ Rebuffering and incubation of this mixture at 98 °C for 1.5–5 h effected hydrolysis of **Rib1P** and gave new equilibrium positions of 54–69 % after additional pH adjustment and addition of fresh nucleoside phosphorylase (see Figure 3 for exemplary data for pH 8). Consistent with the fundamental assumptions and the predictions of equation (12), the new equilibrium position approached but did not surpass 72 % conversion, which would correspond to full hydrolysis of the initially generated **Rib1P**. Calculation of the amount of hydrolyzed **Rib1P** via equation (11) and fitting of the time‐dependent decrease of remaining **Rib1P** as a first order exponential decay yielded half‐lives of 1.8–11.7 h under these conditions (Figures 3, S1 and S2). These results strongly support the observation that **Rib1P** is quite tolerant to high temperatures, with alkaline pH values promoting additional stability.^[12]^ Considering the significant differences in experimental conditions, our derived first‐order rate constants of hydrolysis are comparable to those reported by Bunton and Humeres^[12]^ more than 50 years ago (Figure S3) and reveal that the exponential decrease of the rate constant over pH extends well into the alkaline region, up to at least pH 9. One may expect that the half‐life of **Rib1P** under moderate conditions far exceeds the values we obtained at 98 °C (presumably following Eyring relationships), suggesting that hydrolysis of this compound is a negligible factor in most scenarios employing neutral or alkaline pH values and moderate temperatures. However, in applications where harsher conditions or acidic media are applied, hydrolysis of **Rib1P** should certainly be taken into consideration.

**Figure 3:**
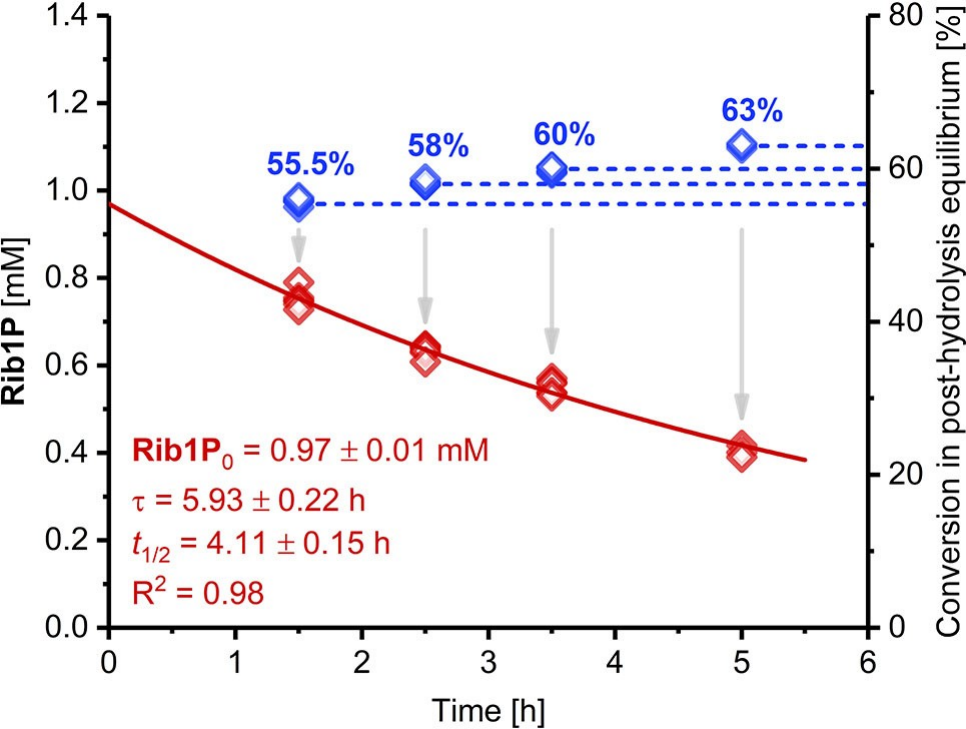
Hydrolysis of **Rib1P** at pH 8 and 98 °C as quantified by phosphorolysis equilibrium shifts. **Rib1P** was generated enzymatically *in situ* through phosphorolysis of uridine catalyzed by *Geobacillus thermoglucosidasius* pyrimidine nucleoside phosphorylase and then subjected to hydrolyzing conditions. Hydrolyzed **Rib1P** after various incubation times was calculated via equations (11) and (S23) from post‐hydrolysis equilibrium positions (blue) and residual **Rib1P** (red) was fitted was a first‐order exponential decay (equation (S23) in the Supplementary Information). Please see the Supporting Information for more experimental details and the externally hosted Supplementary Information for all raw and calculated data.^[18]^

In conclusion, we outlined and demonstrated a tool for the assessment of the stability of pentose‐1‐phosphates based on apparent equilibrium shifts of a nucleoside phosphorolysis reaction. Our results reveal that spectral unmixing‐based reaction monitoring can be used in conjunction with equilibrium thermodynamics to uncover the hydrolysis kinetics of relatively stable sugar phosphates such as ribose‐1‐phosphate. We anticipate that this approach can be extended to other sugar phosphates and related reaction systems where similar effects can be observed.

## Experimental Section

**Rib1P** was generated *in situ* via phosphorolysis of 2 mM uridine with 8 mM potassium phosphate catalyzed by *Geobacillus thermoglucosidasius* pyrimidine nucleoside phosphorylase. Reaction mixtures in equilibrium were aliquoted into PCR tubes and diluted with a buffer mix for pH adjustment. These mixtures were incubated at 98 °C for various times and stored at 4 °C until analysis. To determine the amount of hydrolyzed **Rib1P**, further phosphorolysis of this mixture was initiated by the addition of fresh nucleoside phosphorylase and buffer. This second phosphorolysis reaction was run into its equilibrium and samples were withdrawn and quenched in 100 mM aqueous NaOH. Aliquots of these alkaline samples were transferred to an UV/Vis‐transparent 96‐well plate and UV absorption spectra were recorded from 250 to 350 nm. The respective degree of conversion of the nucleoside to the nucleobase was then obtained via deconvolution of the experimental UV absorption spectra using suitable reference spectra.[^15^, ^16^] Please see the Supporting Information for all experimental details. All raw data presented in this report, along with metadata and calculations are freely available from an external online repository.^[18]^ Likewise, reference spectra and software for spectral unmixing are available from the same repository[^19^, ^20^] and detailed in previous works.[^15^, ^16^]

## Supporting information

As a service to our authors and readers, this journal provides supporting information supplied by the authors. Such materials are peer reviewed and may be re‐organized for online delivery, but are not copy‐edited or typeset. Technical support issues arising from supporting information (other than missing files) should be addressed to the authors.

Supplementary
