# Supplementary material for: Kinetic Analysis of the Hydrolysis of Pentose‐1‐phosphates through Apparent Nucleoside Phosphorolysis Equilibrium Shifts
Source: Chemphyschem. 2020 Dec 4;22(3):283–7. doi: 10.1002/cphc.202000901 (PMC7898831; doi:10.1002/cphc.202000901)
Supplement: Supplementary file 1 — Supplementary [file CPHC-22-283-s001.pdf]

ChemPhysChem

Supporting Information

Kinetic Analysis of the Hydrolysis of Pentose-1-phosphates through Apparent Nucleoside Phosphorolysis Equilibrium Shifts**

Felix Kaspar,* Peter Neubauer, and Anke Kurreck*

Author Contributions

F.K. Conceptualization:Lead; Data curation:Lead; Formal analysis:Lead; Investigation:Lead; Methodology:Lead; Project administration:Equal; Visualization:Lead; Writing – original draft:Lead; Writing – review & editing:Equal
P.N. Funding acquisition:Equal; Resources:Equal; Supervision:Equal; Writing – review & editing:Equal
A.K. Funding acquisition:Equal; Project administration:Equal; Resources:Equal; Supervision:Lead; Writing – review & editing:Equal

Author contributions 1
Data availability 1
Conflict of interest 1
General remarks 2
Experimental 2
Equations 4
Supplementary items 8
Supplementary references 10

Author contributions (with definitions as recommended by Brand *et al.*[1])

Conceptualization, F.K.; Data curation, F.K.; Formal analysis, F.K.; Funding acquisition, P.N. and A.K.; Investigation, F.K.; Methodology, F.K.; Project administration, F.K. and A.K.; Resources, P.N. and A.K.; Software, - ; Supervision, P.N. and A.K.; Validation, - ; Visualization, F.K.; Writing—original draft, F.K.; Writing—review & editing, F.K., P.N. and A.K.

Data availability

All data depicted visually in the items in the main text (Figures 1−3) as well as in the Supplementary Information (Figures S1−3, see below) is available as tabulated data from the externally hosted Supporting Information at zenodo.org.[2]

Conflict of interest

A. K. is CEO of the biotech company BioNukleo GmbH. F. K. is a scientist at BioNukleo GmbH and P. N. is a member of the advisory board. These affiliations constitute no conflict of interest with the results presented and discussed in this report.

General remarks

All chemicals used in this study were of analytical grade or higher and purchased from Sigma Aldrich (Steinheim, Germany), Carbosynth (Berkshire, UK), Carl Roth (Karlsruhe, Germany), TCI Deutschland (Eschborn, Germany) or VWR (Darmstadt, Germany) and used without prior purification. Water deionized to 18.2 MΩ·cm with a Werner water purification system was used for the preparation of all enzymatic reactions as well as purification and storage buffers. For the preparation of NaOH solutions for quenching, deionized water was used. All experiments were carried out in duplicate.

Experimental

Geobacillus thermoglucosidasius pyrimidine nucleoside phosphorylase (*Gt*PyNP) was obtained via heterologous expression in *E. coli* as described recently.[3,4] Briefly the enzyme was heterologously expressed in *E. coli* as His_6-tagged proteins through IPTG-induced overexpression. Purification was achieved through cell disruption, heat treatment of the crude extract (60 °C for 30 min) and Ni-NTA affinity chromatography. Proteins were eluted with buffer containing 250 mM imidazole, 50 mM sodium phosphate, 300 mM NaCl (pH 8) and desalted into 2 mM potassium phosphate buffer (pH 7). The protein was stored as a stock solution of 1.2 g L^{-1} (calculated with 1 AU cm^{-1} at 280 nm being equal to a protein concentration of 1 g L^{-1}) at −20 °C in 50% (v/v) glycerol. Note: We used *Gt*PyNP in our study because that was what we happened to have on hand and, having used this enzymes in previous studies,[5,6] knew about its robustness and performance. In principle, any nucleoside phosphorylase can be employed for the first and second phosphorolysis steps in the described approach. However, this may necessitate the application of different working spaces and consideration of the temperature-dependence of the equilibrium constant of phosphorolysis.[7]

Rib1P was generated *in situ* via enzymatic phosphorolysis of 2 mM uridine with 8 mM potassium phosphate catalyzed by 5 µg mL^{-1} *Gt*PyNP in 10 mM MOPS buffer pH 7 at 50 °C in a total volume of 1.3 mL in an Eppendorf tube. This yielded 1.02 mM **Rib1P** in the equilibrium, corresponding to 50.8% conversion of the nucleoside to the nucleobase. Under these conditions, the equilibrium was reached after ca. 20 min and the reaction was run for an additional 20 min (40 min total) to ensure that the equilibrium was truly established (please also note that the concentrations chosen for this experiment are arbitrary and not relevant – the experiment would have worked the same way with higher or lower concentrations of all components). The resulting reaction mixture was then aliquoted into PCR tubes and diluted with a buffer mix for pH adjustment (150 µL reaction mixture + 50 µL buffer stock) to achieve final concentrations of 0.75 mM **Rib1P**, 5 mM citrate, 17.5 mM MOPS and 20 mM glycine at pH 7−9. These mixtures were transferred to a PCR cycler and incubated at 98 °C (with 105 °C lid temperature to prevent evaporation). At various points in time, tubes were removed from the PCR cycler and stored at 4 °C until analysis. A second phosphorolysis step was initiated to determine the

amount of hydrolyzed **Rib1P**. To this end, further phosphorolysis of this mixture was started by the addition of fresh *Gt*PyNP and MOPS buffer pH 7 to reach final concentrations of 5 µg mL^{-1} and 50 mM, respectively (150 µL incubation mix + 50 µL enzyme and buffer mix containing 20 µg mL^{-1} *Gt*PyNP and 50 mM MOPS buffer). The resulting mixture contained 0.56 mM ribosyl compound (**Rib1P** and ribose), 3.8 mM citrate, 63.1 mM MOPS, 15 mM glycine and 5 µg mL^{-1} *Gt*PyNP. This second phosphorolysis reaction was run into its equilibrium and samples of 40 µL were withdrawn and quenched in 300 µL 100 mM aqueous NaOH after 20, 30 and 40 min. The dilution steps described above effected a decrease in concentration of all initial reaction components. Since all initial components (nucleoside, base, phosphate, **Rib1P**) were diluted equally with each step, this dilution factor can be neglected since our chosen analytical method[4,8] records molar ratios of the nucleoside and the nucleobase and derived conversions are relative. Using a concentration-independent method to determine the concentration allowed direct extrapolation of the relative amounts observed after dilution to the relative amounts present in the initial reaction.

Reaction monitoring was achieved via spectral unmixing. From reactions in equilibrium, samples of 40 µL were withdrawn and quenched in 300 µL 100 mM aqueous NaOH as described previously.[4,8] Of these diluted alkaline samples, 200 µL were transferred to UV/Vis-transparent 96-well plates (UV star, GreinerBioOne, Kremsmünster, Austria) for analysis. UV absorption spectra were recorded from 250−350 nm with a BioTek PowerWave HT platereader (BioTek Instruments, Winooski, USA) and subjected to spectral unmixing using analogously obtained reference spectra. Reference spectra used in this study are freely available in the externally hosted Supplementary Information and can, alternatively, be obtained from the Supplementary Information of previous publications.[9] The degree of conversion was determined directly from the spectra fit which considers the UV-active substrate and product in relation to one another.[4] All experimental spectra were fit to the information-rich shoulder region of the uridine-uracil pair (270−300 nm) and corrected for time- and condition-depended background spectra as detailed in the metadata files in the externally hosted supplementary information.

Equations

The following describes the derivation of the equations given in the main text and further equations and assumptions used for calculation. For equations appearing in the main text, the equation numbers as listed in the main text were adopted, even if their order in this document may not be identical. All other equations were listed in order as supplementary equations (S1)–(S26).

The initial phosphorolysis reaction can be described by the law of mass action

$$K = \frac{[P1P][B]}{[N][P]} \tag{1}$$

with the initial mass balances

$$[P1P] = [B] = [P1P]_1 = [B]_1 \tag{2}$$

$$[N] = [N]_0 - [B]_1 \tag{3}$$

$$[P] = [P]_0 - [B]_1 \tag{4}$$

where K is the equilibrium constant of phosphorolysis and $[P1P]$, $[B]$, $[N]$ and $[P]$ are the equilibrium concentrations of the pentose-1-phosphate, nucleobase, nucleoside and phosphate, respectively, and $[P1P]_1$ and $[B]_1$ are the concentrations of the pentose-1-phosphate and the nucleobase and the first (pre-hydrolysis) equilibrium and $[N]_0$ and $[P]_0$ are the initial concentrations of the nucleoside and phosphate. It is herein assumed that the initial concentrations of the products are equal to zero and the reaction generally adheres to stoichiometry (i.e. consumption of 1 mol nucleoside and 1 mol phosphate produced 1 mol nucleobase and 1 mol pentose-1-phosphate). The degree of conversion of the nucleoside to the nucleobase in this first equilibrium c_1 is given as

$$c_1 = \frac{[B]_1}{[N]_0} \tag{5}$$

Termination of the enzymatic reaction (e.g. through short boiling of the reaction mixture) prevents further phosphorolysis of the nucleoside. When this mixture is then subjected to hydrolyzing conditions, a fraction (or all) of the provided pentose-1-phosphate will by hydrolyzed into ribose and phosphate, giving the new mass balances

$$[P1P] = [B]_1 - [P1P]_h \tag{S1}$$

$$[P] = [P]_0 - [B]_1 + [P1P]_h \tag{S2}$$

where $[P1P]_h$ is the amount of hydrolyzed pentose-1-phosphate. The mass balances of the nucleoside and the nucleobase remain identical. This process yields a reaction mixture containing the nucleoside and nucleobase concentration established by the initial phosphorolysis with the phosphate and pentose-1-phosphate concentrations depending on the extent of hydrolysis of the latter. At this point, continuation of phosphorolysis (through addition of fresh enzyme) will establish a new equilibrium to account for the loss of pentose-1-phosphate and the increase of phosphate compared to its phosphorolysis equilibrium concentration. Therefore, continued conversion of the nucleoside to the

nucleobase and pentose-1-phosphate will be observed until a new equilibrium is reached, which again complies with the law of mass action given in equation (1). The resulting extended mass balances in this new equilibrium can be formulated as

$$[P1P] = [B]_1 - [P1P]_h + [B]_n \tag{S3}$$

$$[B] = [B]_1 + [B]_n \tag{S4}$$

$$[N] = [N]_0 - [B]_1 - [B]_n \tag{S5}$$

$$[P] = [P]_0 - [B]_1 - [B]_n + [P1P]_h \tag{S6}$$

where $[B]_n$ is the concentration of newly consumed nucleobase. Since conversion of the nucleoside to the nucleobase is easily measured in relation (i.e. molar ratios), for example by spectral unmixing,[4,8] the degree of conversion in these equilibria can be employed to simplify these equations by assuming that

$$[B]_1 = c_1[N]_0 \tag{S7}$$

$$[B]_n = [N]_0(c_2 - c_1) \tag{S8}$$

where c_1 is the degree of conversion of the nucleoside to the nucleobase in the first equilibrium as given in equation (5) and c_2 is the degree of conversion in the second equilibrium defined as

$$c_2 = \frac{[B]_2}{[N]_0} \tag{10}$$

These degrees of conversion are equivalent to the relative molar amounts of the nucleobase compared to the nucleoside (i.e. a c_1 = 0.1 is equivalent to 10% conversion of the nucleoside to the nucleobase). Substitution in the above equations gives the mass balances

$$[P1P] = c_1[N]_0 - [P1P]_h + [N]_0(c_2 - c_1) \tag{S9}$$

$$[B] = c_1[N]_0 + [N]_0(c_2 - c_1) \tag{S10}$$

$$[N] = [N]_0 - c_1[N]_0 - [N]_0(c_2 - c_1) \tag{S11}$$

$$[P] = [P]_0 - c_1[N]_0 - [N]_0(c_2 - c_1) + [P1P]_h \tag{S12}$$

which can be simplified by collapsing all terms containing c_1 to yield

$$[P1P] = c_2[N]_0 - [P1P]_h \tag{6}$$

$$[B] = c_2[N]_0 \tag{7}$$

$$[N] = [N]_0 - c_2[N]_0 \tag{8}$$

$$[P] = [P]_0 - c_2[N]_0 + [P1P]_h \tag{9}$$

To determine the quantity of interest, $[P1P]_h$, the equations (5)–(8) can be inserted into the law of mass action (1), giving

$$K = \frac{(c_2[N]_0 - [P1P]_h)c_2[N]_0}{([N]_0 - c_2[N]_0)([P]_0 - c_2[N]_0 + [P1P]_h)} \tag{S13}$$

which can be rewritten as

$$K = \frac{c_2^2[N]_0^2 - [P1P]_h c_2[N]_0}{[N]_0[P]_0 - c_2[N]_0^2 + [N]_0[P1P]_h - c_2[N]_0[P]_0 + c_2^2[N]_0^2 - c_2[N]_0[P1P]_h} \tag{S14}$$

Solving this expression for $[P1P]_h$ via

$$\begin{aligned} K[N]_0[P]_0 - Kc_2[N]_0^2 + K[N]_0[P1P]_h - Kc_2[N]_0[P]_0 + Kc_2^2[N]_0^2 - Kc_2[N]_0[P1P]_h \\ - c_2^2[N]_0^2 = -[P1P]_h c_2[N]_0 \end{aligned} \tag{S15}$$

and

$$\begin{aligned} K[N]_0[P]_0 - Kc_2[N]_0^2 - Kc_2[N]_0[P]_0 + Kc_2^2[N]_0^2 - c_2^2[N]_0^2 \\ = [P1P]_h(Kc_2[N]_0 - c_2[N]_0 - K[N]_0) \end{aligned} \tag{S16}$$

gives

$$[P1P]_h = \frac{K[N]_0[P]_0 - Kc_2[N]_0^2 - Kc_2[N]_0[P]_0 + Kc_2^2[N]_0^2 - c_2^2[N]_0^2}{[N]_0(Kc_2 - c_2 - K)} \tag{S17}$$

simplified to

$$[P1P]_h = \frac{K([P]_0 - c_2[N]_0 - c_2[P]_0 + c_2^2[N]_0) - c_2^2[N]_0}{Kc_2 - c_2 - K} \tag{11}$$

The expected maximum conversion in the second equilibrium $c_{2,max}$ can be obtained through the assumption of full hydrolysis of the initially generated sugar phosphate

$$[P1P]_h = c_1[N]_0 \tag{S18}$$

Substitution in equation (9) gives

$$c_1[N]_0 = \frac{K\left([P]_0 - c_{2,max}[N]_0 - c_{2,max}[P]_0 + c_{2,max}^2[N]_0\right) - c_{2,max}^2[N]_0}{Kc_{2,max} - c_{2,max} - K} \tag{S19}$$

which can be rearranged to

$$\begin{aligned} c_1[N]_0\left(Kc_{2,max} - c_{2,max} - K\right) = \\ K\left([P]_0 - c_{2,max}[N]_0 - c_{2,max}[P]_0 + c_{2,max}^2[N]_0\right) - c_{2,max}^2[N]_0 \end{aligned} \tag{S20}$$

to give a quadratic equation where $c_{2,max}$ is a function of K, $[N]_0$, $[P]_0$ and c_1. Although c_1 itself is a function of K, $[N]_0$ and $[P]_0$, insertion of its analytical solution (equation (4) in [7]) would tremendously complication this term which is why, for the sake of simplicity, we herein opted to keep c_1 in this expression. Equation (S20) can then be further rearranged to

$$\begin{aligned} c_1[N]_0Kc_{2,max} - c_1[N]_0c_{2,max} - c_1[N]_0K - K[P]_0 + Kc_{2,max}[N]_0 + Kc_{2,max}[P]_0 \\ - Kc_{2,max}^2[N]_0 + c_{2,max}^2[N]_0 = 0 \end{aligned} \tag{S21}$$

and

$$\begin{aligned} c_{2,max}^2([N]_0 - K[N]_0) + c_{2,max}(c_1[N]_0K - c_1[N]_0 + [N]_0K + K[P]_0) - c_1[N]_0K \\ - K[P]_0 = 0 \end{aligned} \tag{S22}$$

with the solution

$$c_{2,max} = \frac{-X + \sqrt{X^2 + 4([N]_0 - K[N]_0)(c_1[N]_0K + K[P]_0)}}{2[N]_0(1 - K)} \tag{12}$$

where

$$X = c_1[N]_0K - c_1[N]_0 + [N]_0K + K[P]_0 \tag{13}$$

The maximum apparent equilibrium shift Δc_{max} is therefore a function of $[N]_0$, $[P]_0$ and K and can be obtained via the above equations through

$$\Delta c_{max} = c_{2,max} - c_1 \tag{14}$$

Since water as a reagent for the hydrolysis of pentose-1-phosphates is present in excess, this reaction most likely proceeds via first-order kinetics where the decrease of the pentose-1-phosphate concentration can be describes by

$$[P1P](t) = [P1P]_0 \exp\left(-\frac{t}{\tau}\right) = [P1P]_0 - [P1P]_h \tag{S23}$$

where $[P1P]_0$ is the concentration of the pentose-1-phosphate in the first equilibrium (before hydrolysis), t is the incubation time and τ is the mean half-life. The decay constant k is then available via

$$k = \tau^{-1} \tag{S24}$$

and the half-life $t_{1/2}$ can be obtained through

$$t_{1/2} = \tau\, ln(2) \tag{S25}$$

The temperature-dependence of the rate constant k likely follows the Eyring equation[10]

$$k = \frac{\kappa k_b T}{h} \exp\left(-\frac{\Delta_R G^{\ddagger}}{RT}\right) \tag{S26}$$

where k_{obs} is the observed rate constant [s^{-1}], κ is the transmission coefficient (herein assumed to be unity), k_b is the Boltzmann constant (1.38 · 10^{-23} J K^{-1}), T is the temperature [K], h is the Planck constant (6.626 · 10^{-34} J s), R is the universal gas constant (8.314 J mol^{-1} K^{-1}), $\Delta_R G^{\ddagger}$ the transition state free enthalpy [J mol^{-1}].

Supplementary figures

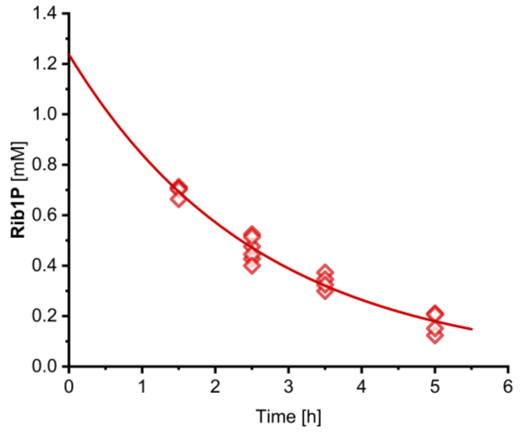

Figure S1. Hydrolysis of **Rib1P** at 98 °C and pH 7. The fit of the experimental data yielded τ = 2.59 ± 0.15 h and $[P1P]_0$ = 1.23 ± 0.06 mM (R^2 = 0.96). Please see the externally hosted Supplementary Information for all raw data and calculations.[2]

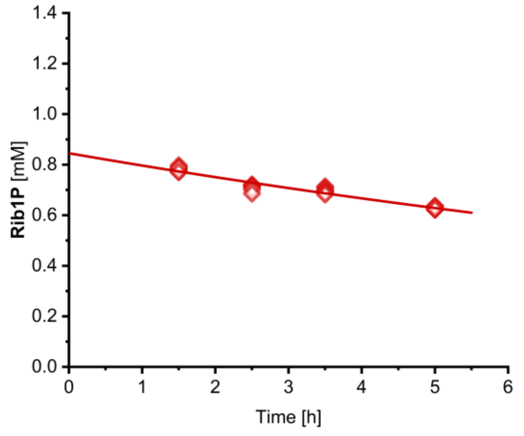

Figure S2. Hydrolysis of **Rib1P** at 98 °C and pH 9. The fit of the experimental data yielded τ = 16.87 ± 1.07 h and $[P1P]_0$ = 0.85 ± 0.01 mM (R^2 = 0.92). Please see the externally hosted Supplementary Information for all raw data and calculations.[2]

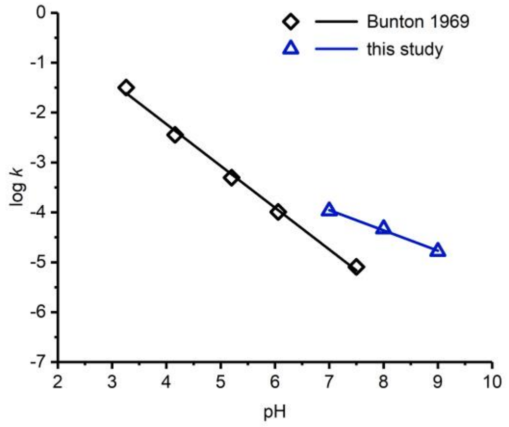

Figure S3. Comparison of the kinetic data of Bunton and Humeres[11] with those obtained in this study. The data of Bunton were extrapolated from 82 °C to 98 °C assuming temperature-independent $\Delta_R G^‡$ values as listed in Table S1. Bunton used diluted acid in their experiments and monitored the hydrolysis of pure **Rib1P** via liberation of phosphate as assessed by a colorimetric assay. This study employed a buffered enzymatic reaction and *in situ* generated **Rib1P** and monitored its hydrolysis through apparent equilibrium shifts of a nucleoside phosphorolysis reaction.

Table S1. Extrapolation of the kinetic data of Bunton and Humeres[11]

pH	k (82 °C) [10^4 s^{-1}][a]	k (82 °C) [s^{-1}]	$\Delta_R G^‡$ [kJ mol^{-1}]	k (98 °C) [s^{-1}]
3.26	68.3	0.00683	102.2	0.03174604
4.16	7	0.0007	108.9	0.00358935
5.2	0.89	0.000089	115.0	0.000498794
6.06	0.17	0.000017	119.9	0.000102323
7.5	0.012	0.0000012	127.7	0.000081

[a] as listed in their Table 1 on page 2 (entries 5−9).

Supplementary references

[1] A. Brand, L. Allen, M. Altman, M. Hlava, J. Scott, *Learn. Publ.* **2015**, *28*, 151–155.

[2] F. Kaspar, **2020**, DOI 10.5281/zenodo.4088390.

[3] K. Szeker, X. Zhou, T. Schwab, A. Casanueva, D. Cowan, I. A. Mikhailopulo, P. Neubauer, *J. Mol. Catal. B Enzym.* **2012**, *84*, 27–34.

[4] F. Kaspar, R. T. Giessmann, N. Krausch, P. Neubauer, A. Wagner, M. Gimpel, *Methods Protoc.* **2019**, *2*, 60.

[5] R. T. Giessmann, N. Krausch, F. Kaspar, N. M. Cruz Bournazou, A. Wagner, P. Neubauer, M. Gimpel, *Processes* **2019**, *7*, 380.

[6] F. Kaspar, P. Neubauer, A. Kurreck, *ChemRxiv* **2020**, DOI 10.26434/chemrxiv.12993416.v1.

[7] F. Kaspar, R. T. Giessmann, P. Neubauer, A. Wagner, M. Gimpel, *Adv. Synth. Catal.* **2020**, *362*, 867–876.

[8] F. Kaspar, R. T. Giessmann, S. Westarp, K. F. Hellendahl, N. Krausch, I. Thiele, M. C. Walczak, P. Neubauer, A. Wagner, *ChemBioChem* **2020**, *21*, 2604.

[9] F. Kaspar, **2020**, DOI 10.5281/zenodo.3716126.

[10] H. Eyring, *J. Chem. Phys.* **1935**, *3*, 107–115.

[11] C. A. Bunton, E. Humeres, *J. Org. Chem.* **1969**, *34*, 572–576.
